# CWN-2/Wnt regulates SMDD axonal development

**DOI:** 10.17912/micropub.biology.000337

**Published:** 2020-11-25

**Authors:** Tessa Sherry, Hannah R Nicholas, Roger Pocock

**Affiliations:** 1 Monash University; 2 University of Sydney

**Figure 1. The Wnt ligand CWN-2 and Ror receptor CAM-1 control SMDD axonal development:**
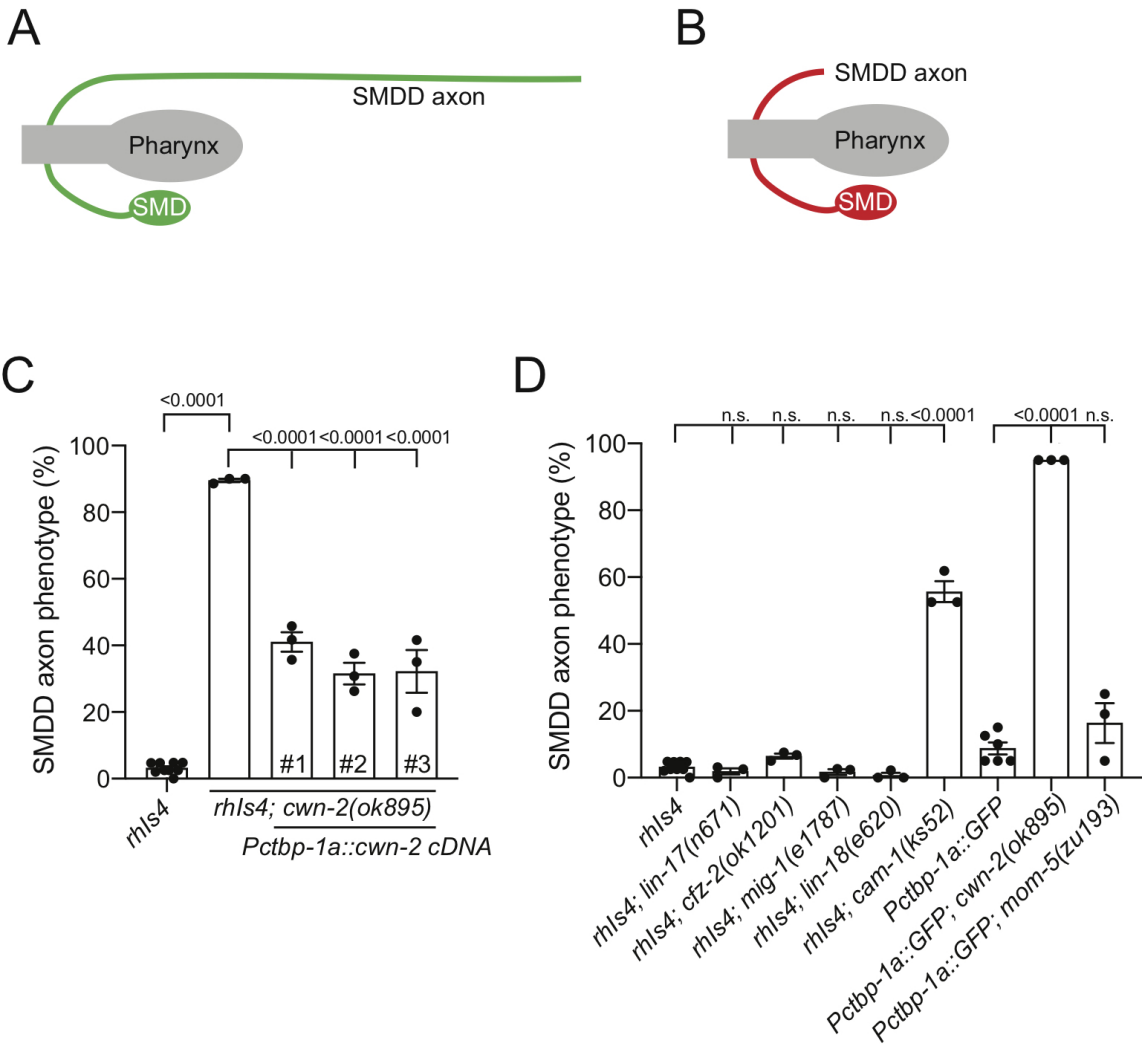
(A-B) Schematic of a wild-type (A) and defective (B) SMDD axon where the axon is not visible in the sublateral nerve cord. (C) Quantification of the SMDD axonal phenotype in the *cwn-2(ok895)* mutant and rescue using three independent transgenic lines expressing *Pctbp-1::cwn-2* cDNA (#1-3). (D) Quantification of the SMDD axonal phenotype of mutant alleles for Wnt receptors LIN-17, CFZ-2, MIG-1,LIN-18, CAM-1 and MOM-5. SMDD development was analyzed with either the *rhIs4(Pglr-1::GFP)* or *Pctbp-1a::GFP* reporter and scored defective if the SMDD axon was not visible in the sublateral nerve cord. Data presented as mean ± S.E.M (bar) of at least three biological replicates (black dots) by one-way ANOVA with Tukey’s correction. n>60 axons per bar. n.s. – not significant.

## Description

CWN-2 is one of five *C. elegans* Wnt ligands, with known roles in neuron migration, axon guidance and nerve ring placement (reviewed in (Sawa and Korswagen, 2013)). CWN-2 is highly expressed in the SMDD neurons (Kennerdell *et al.*, 2009; Taylor *et al.*, 2019), and therefore we were interested in determining whether CWN-2 regulates SMDD axonal development.

We used the *cwn-2(ok895)* deletion allele to analyze SMDD axonal development. We found that loss of *cwn-2* caused a defective SMDD axonal phenotype, where axons do not extend along the dorsal sublateral cord, suggesting that the axons did not exit the nerve ring due to defects in axon outgrowth or guidance (Figure 1A-C). To ensure that this phenotype was due to the loss of CWN-2 function, we transgenically expressed *cwn-2* cDNA driven by the *ctbp-1a* promoter, which drives expression in SMDD and ten other head neurons (Sherry *et al.*, 2020). Expressing *cwn-2* using this promoter partially rescues the defective axonal phenotype (Figure 1C). This suggests that CWN-2 controls SMDD development in an autocrine manner and/or non-autonomously from other head neurons.

Next, we investigated which Wnt receptor controls SMDD axonal development. There are six Wnt receptors encoded in the *C. elegans* genome: four Frizzled receptors (LIN-17, CFZ-2, MIG-1 and MOM-5,), one Ror receptor (CAM-1) and one Ryk receptor (LIN-18) (Sawa and Korswagen, 2013). We analyzed the effect of loss-of-function mutations for each receptor and found that loss of *cam-1,* but not the other receptors, caused defective SMDD axonal development (Figure 1D). CAM-1 was previously shown to function as a CWN-2 receptor for SIA, SIB and RMED/V posterior-directed neurite outgrowth (Kennerdell *et al.*, 2009; Song *et al.*, 2010; Wang and Ding, 2018). The SIA, SIB, RMED/V and SMDD motor neuron cell bodies are all positioned in the nerve ring and extend posteriorly-directed axons (White *et al.*, 1986). Therefore, our data show that CWN-2 and CAM-1 have a common role in controlling axon extension in posteriorly-directed lateral or sublateral axons.

## Methods

*C. elegans s*trains were grown using standard growth conditions on NGM agar at 20 °C and fed with *Escherichia coli* OP50. Neuroanatomical reporter strains used – *rhIs4 [Pglr-1::GFP]* and *rpEx1739[Pctbp-1a::GFP]*. Homozygous *mom-5* mutant animals were selected based on the Unc phenotype caused by the linked *unc-13(e1091)* mutation. Day two adult hermaphrodites were anesthetized with 0.2% levamisole hydrochloride on 5% agarose pads for anatomical scoring. Schematics of axonal defects are shown instead of images as bright GFP expression in multiple cell bodies around the pharynx obscure imaging of axon defects. Refer to (Sherry *et al.*, 2020) for images of the SMDD axons posterior to the pharynx. ‘Defective SMDD axonal phenotype (%)’ indicates the percentage of SMDD axons that do not extend along the dorsal sublateral cord. Two SMDD axons (L and R) were scored per animal. For *cwn-2(ok895)* rescue experiments, transgenic animals were compared to the *cwn-2(ok895)* mutant. Three biological replicates were performed, and statistical significance was assessed by one-way ANOVA followed by Tukey’s multiple comparisons tests.

Cloning was performed using Takara In-Fusion® restriction-free cloning. The *Pctbp-1a::cwn-2* rescue construct was generated by cloning the *cwn-2* 1083 bp cDNA sequence, amplified from *C. elegans* cDNA, into a *Pctbp-1a::GFP* vector to replace the GFP sequence (Sherry *et al.*, 2020). Rescue constructs were injected into *rhIs4; cwn-2(ok895)* mutant backgrounds at 2 ng/μl with *Punc-122::GFP* (20 ng/μl) as injection marker.

## Reagents

**Strains**

HRN169 *rhIs4 [Pglr-1::GFP] III*

HRN498 *rhIs4 III; cwn-2(ok895) IV*

RJP4553 *rhIs4 III; cwn-2(ok895) IV; rpEx2027 [Pctbp-1a::cwn-2]* line #1

RJP4554 *rhIs4 III; cwn-2(ok895) IV; rpEx2028 [Pctbp-1a::cwn-2]* line #2

RJP4555 *rhIs4 III; cwn-2(ok895) IV; rpEx2029 [Pctbp-1a::cwn-2]* line #3

RJP4485 *mig-1(e1787) I; rhIs4 III*

RJP4486 *lin-17(n671) I; rhIs4 III*

RJP4534 *rhIs4 III; cfz-2(ok1201) V*

RJP4688 *cam-1(ks52) II; rhIs4 III*

RJP4715 *rhIs4 III; lin-18(e620) X*

RJP4076 *rpEx1739 [Pctbp-1a::GFP]*

RJP4598 *cwn-2(ok895) IV; rpEx1739 [Pctbp-1a::GFP]*

RJP4624 *mom-5(zu193) unc-13(e1091)/hT2 III; rpEx1739 [Pctbp-1a::GFP]*
